# Usability of an App for Medical History Taking in General Practice From the Patients’ Perspective: Cross-Sectional Study

**DOI:** 10.2196/47755

**Published:** 2024-01-05

**Authors:** Klara Albrink, Dominik Schröder, Carla Joos, Frank Müller, Eva Maria Noack

**Affiliations:** 1 Department of General Practice University Medical Center Göttingen Göttingen Germany

**Keywords:** digitization, application software, usability, mHealth, history of present illness, medical history taking

## Abstract

**Background:**

A future shortage of physicians, especially in general practice, will result in an increasing workload for health care providers as a whole. Therefore, it is important to optimize patient-encounter processes to increase time efficiency related to visits. Utilizing digital tools to record patients’ medical histories prior to a consultation offers great potential to achieve this goal. The collected information can be stored into the practice’s electronic medical record, allowing for the general practitioner to review structured information of the patients’ complaints and related medical history beforehand, thereby saving time during the encounter. However, the low usability of new digital developments in this setting often hinders implementation.

**Objective:**

The aim of this study was to evaluate the usability of an app designed for medical history taking in general practice to capture the patients’ perspective.

**Methods:**

Between November 2021 and January 2022, we recruited 406 patients with acute complaints in one out-of-hour urgent care and seven general practice clinics. These study participants used the app during their waiting time and subsequently assessed its usability by completing the System Usability Scale (SUS), a robust and well-established 10-question survey measuring the perceived usability of products and technologies. Additionally, we collected general participant information, including age, sex, media usage, health literacy, and native language. Descriptive and inferential statistics were applied to identify patient characteristics associated with low or high SUS scores.

**Results:**

We analyzed data from 397 patients (56.7% female, 43.3% male). The mean total SUS score was 77.8 points; 54.4% (216/397) of participants had SUS scores of 80 points or higher, indicating high usability of the app. In a multiple linear regression predicting SUS score, male sex and higher age (65 years or older) were significantly negatively associated with the SUS score. Conversely, a higher health literacy score and German as the native language were significantly positively associated with the SUS score.

**Conclusions:**

Usability testing based on the SUS anticipates successful implementation of the app. However, not all patients will easily adapt to utilizing the app, as exemplified by the participants of older age in this study who reported lower perceived usability. Further research should examine these groups of people, identify the exact problems in operating such an app, and provide targeted solutions.

**Trial Registration:**

German Clinical Trials Register World Health Organization Trial Registration Data Set DRKS00026659; https://trialsearch.who.int/Trial2.aspx?TrialID=DRKS00026659

## Introduction

As in many countries, demographic change is becoming evident in the German health care system, resulting in more complex, multimorbid patients [1] and a shortage of physicians [2]. Moreover, the proportion of older people in the population is rising steadily [3] and people tend to use medical services at a higher rate as they increase in age [4]. In Germany, one group that is particularly affected by this development are general practitioners (GPs) who are the first point of contact for patients requiring medical care and serve as the “gatekeepers” in the German health care system [5]. Approximately 80% of the German population aged 18 years and older are treated by a GP at least once a year [6]. A considerable number of GPs will retire in the upcoming years, resulting in 11,000 GP vacancies expected by 2035. These vacancies will disproportionately impact structurally weak and rural areas [7,8]. Without a sufficient workforce to replace the retired GPs and meet the greater demand for physicians, remaining GP workloads are expected to increase significantly within the next decade [9]. These developments challenge the German health care system at various levels and require attention to address the following key issues: future financing, improving allocation of resources, ensuring access to care, increasing efficiency and effectiveness of health care provision, and strengthened collaboration between providers [10].

To streamline patient care in the upcoming years, it is of importance to optimize patient-encounter processes to increase time efficiency related to visits. In this respect, digital tools offer great potential to support GPs in patient management, documentation workload, and the collection of medical history before consultation.

Digital tools designed to collect patients’ medical history can ensure that information is always collected and documented thoroughly in a structured manner and with consistent quality. As many conditions can be diagnosed via a thorough medical history [11,12], these tools can be helpful in maintaining quality of care when time constraints may lead to an otherwise superficial medical history.

As part of our project titled “Digitally assisted information acquisition before medical consultation” (DASI), we developed an app for medical history taking in general practice settings. The app is used by the patient prior to the medical consultation, which could be either in the waiting area or at home. The collected information can be stored in the practice’s electronic medical record and eventually be transferred to the individual electronic patient file, which statutory health insurers in Germany have been entitled to use since 2021 [13]. In the electronic patient file, patient data such as medical reports, X-rays, immunization records, and other medical data can be stored and shared among medical providers involved in the care of a particular patient by using the telematics infrastructure [14].

One advantage of the app is that the GP can review structured information of the patients’ complaints and related medical history before the encounter. This is particularly helpful for patients that are unknown to the provider, those with many complaints, or those who have a comprehensive medical history. These situations are especially prevalent in out-of-hour urgent care practices. Furthermore, the tool might help patients to reflect on their conditions and enable them to better address their needs when seeing the provider. In this way, we expect that the limited consultation time can be used more efficiently.

Despite Germany’s progress in digitalization within the health sector, concerns remain about the limited usability of new digital tools, hindering their full implementation. More than half of German practices see low usability as a strong obstacle to digitalization [15]. The evaluation of a digital tool’s perceived usability is of special interest as it is a key determinant of performance for end users. Therefore, the aim of this study was to assess the usability of the app from the patients’ perspective and to identify features in need of improvement. The broader aim is to ensure that the app is suitable for implementation in everyday practice, considering that GPs treat a broad range of patients of all ages and various educational and cultural backgrounds [16].

## Methods

### Study Design and Recruitment

This was a cross-sectional study conducted in Germany in one out-of-hour urgent care practice and seven GP practices to assess the usability of an app designed for medical history taking in general practice settings.

### Software and Hardware

The app was developed to take a medical history based on general medical complaints directly from the patients. While there are no international standards for the composition of a standardized patient history, this app was developed based on guidelines and health literature by medical experts from aidminutes GmbH (Hamburg/Buchholz in der Nordheide, Germany). For this study, the content and query structure were further refined for primary care (general practice and out-of-hour practices) by aidminutes GmbH in collaboration with experienced researchers from the Department of General Practice at the University Medical Center Göttingen, Germany. The app was designed to be used by patients in the waiting room before they see the doctor. Patients select one or several complaints and are then guided through a symptom-related questionnaire. In the sense of a branching logic, the app is adaptive to patient responses, which trigger further specific questions about the selected key complaints (eg, how and when a symptom started). Patients are also asked about preexisting conditions, previous treatments and surgeries, current medication, living habits, and chronic conditions in the family history. Information such as biological sex, height, weight, age, as well as the subjectively perceived severity of the complaints are inquired from all patients. More details can be found in the published study protocol [17].

**Figure 1:**
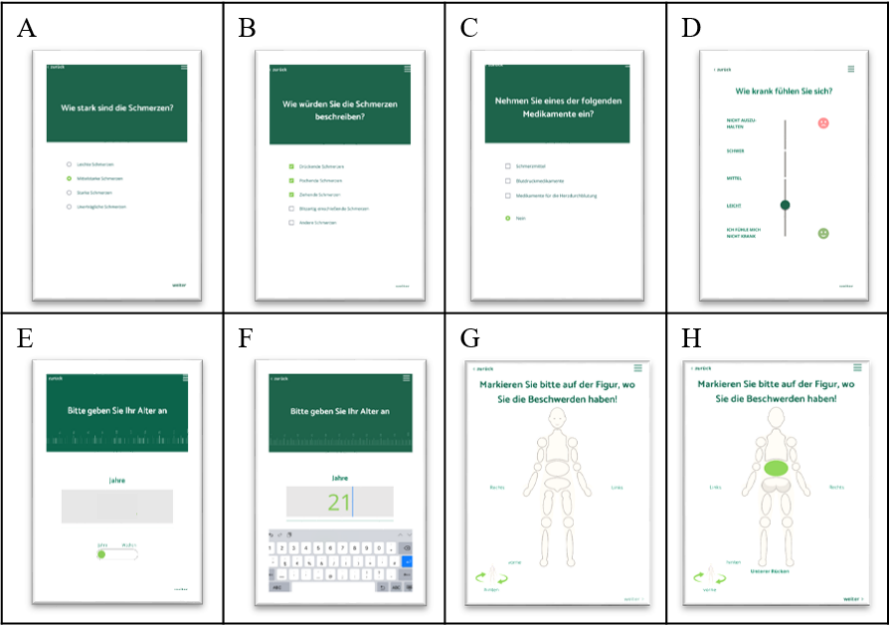
Screenshots of the app for medical history taking in general practice showing different types of questions: (A) single-choice question; (B) multiple-choice question; (C) hybrid question (ie, patients can either select several options or negate all of them); (D) slider for questions including a ranking between items (depicted here as “How sick do you feel?”); (E, F) data entry field (here: “Please enter your age”); and (G, H) selection of a body region on a figure (depicted in figure: “Please mark on the figure where you are suffering from the problems”).

The app was designed to be intuitive for the user such that no prior knowledge or any kind of instruction for its use is necessary. The user interface was designed to be simple to follow and only one question is asked per screen. As the app is operated in the waiting area, sound and video output of an earlier version [18] was omitted due to data protection. The questions are phrased in plain language; medical terminology is avoided or otherwise explained. The questions are substantially comprised by single-choice or multiple-choice questions that can be answered by tapping but also include several data fields (for age, height, and weight) and slider-type questions (Figure 1). The color scheme was designed to ensure reading accessibility for patients who may be color blind. A zoom function can be used for users who may experience visual impairment.

As this is a web-based app, it relies on a permanent internet connection. For this study, the app ran on an iPad Mini 4 (Apple Inc, Cupertino, CA, USA) held in an upright position. Tablets were equipped with haptically and visually inconspicuous cases (dark grey polyurethane leather outside and microfiber inside).

### Setting

In Germany, GP practices aim at providing preventive, acute, and rehabilitative health care with long-lasting patient-doctor relationships. Out-of-hours urgent care practices provide urgent medical care for acute but not life-threatening cases when other practices are closed. Urgent care practices are often staffed with doctors of various specialties and an established relationship of care between the patients and doctors is not common. These aspects can lead to challenges in efficiently obtaining an accurate medical history and identifying serious health problems. Although the app was designed for general practice, it is also suitable to be used in out-of-hour urgent care practices.

### Data Collection

The recruitment of patients was carried out by three study nurses and took place from November 22, 2021, to January 12, 2022. Patients were approached by study nurses in the waiting room of the respective practices before seeing their GP.

Patients meeting the following criteria were eligible to participate in the study: (1) seeking care in a participating practice because of acute somatic and/or psychological complaints, (2) at least 18 years old, and (3) consenting to participate in the study. Patients meeting the following exclusion criteria could not participate in the study: (1) younger than 18 years old (legal minor), (2) patients in an apparent emergency, (3) patients who required immediate medical treatment, and (4) patients who were unable to provide consent.

After the study nurses obtained written informed consent, a tablet on which the app was run on was handed over to the study participants. Participants used the app to report their medical history without an introduction on how to navigate the app. Once finished, they were asked to answer questions on personally perceived usability, media usage, and further sociodemographic data, which were digitally attached to the medical history–taking document. The study nurse in charge was present to observe any problems study participants may have had with using the app and was available to answer questions about the app’s content and usability if specifically requested. Data were collected in an anonymized format without any personal information (eg, name or address) linking the results to each study participant. More detailed information on the data collection can be found in the study protocol [17].

### Ethical Considerations

The Medical Ethics Committee of the University Medical Center Göttingen approved the study (approval number 26/3/21). A written informed consent form was collected from all patients before their inclusion in the study. Participating in the study was voluntary for patients. Patients could withdraw from participation without giving a reason at any time before they had completed the survey. Subsequently, their data could no longer be deleted because it could not be traced back to the individual.

### Measures

The main outcome “usability” was measured using the System Usability Scale (SUS) [19], a commonly used instrument for this purpose [20]. The SUS was developed based on Standard ISO 9241-11 [21], in which usability is measured by the three main attributes of “effectiveness,” “efficiency,” and “satisfaction” [22,23]. Compared to other instruments, the SUS offers several advantages: (1) it can be analyzed quickly, (2) it is relatively easy to understand by academics from other disciplines [24], (3) it contains only 10 statements for easy completion, and (4) it can be used to evaluate almost any type of user interface [25]. We used the translated and validated German version of the SUS [26] and modified the statements to suit our purpose (see Multimedia Appendix 1).

The SUS consists of 10 statements (Table 1), where statements 1, 3, 5, 7, and 9 are positively connoted and statements 2, 4, 6, 8, and 10 are negatively connoted [19]. The scores for these statements are therefore inverted when calculating the sum. The raters decide on the extent to which they agree or disagree to these statements on a 5-point Likert scale ranging from 0 (strongly disagree) to 4 (strongly agree). The final sum score is multiplied by 2.5, resulting in a score range of 0-100 with higher scores indicating better usability [19].

Lewis and Sauro [27] developed a curved grading scale for SUS scores by comparing more than 200 industrial usability studies and using the percentile ranges, resulting in grades “C” (scores of 62.7-72.5), “B” (scores of 72.6-78.8), and “A” (scores of 78.9-100). As a SUS score of 80 proves an above-average user experience, it has become a common industrial goal. This threshold was therefore used for interpreting our results.

**Table 1:** Items of the System Usability Scale (SUS) [19].

Items	English version of the statement
SUS 1	I think that I would like to use this system frequently
SUS 2^a^	I found the system unnecessarily complex
SUS 3	I thought the system was easy to use
SUS 4^a^	I think that I would need the support of a technical person to be able to use this system
SUS 5	I found the various functions in this system were well integrated
SUS 6^a^	I thought that there was too much inconsistency in this system
SUS 7	I would imagine that most people would learn to use this system very quickly
SUS 8^a^	I found the system very cumbersome to use
SUS 9	I felt very confident using the system
SUS 10^a^	I needed to learn a lot of things before I could get going with this system

^a^The scores of negatively connoted SUS items were inverted when calculating the sum.

### Covariates

Consultations in general practice are attended by patients of different ages and educational as well as cultural backgrounds, who have a different quantities of digital interactions in everyday life. To determine whether these factors have an influence on the personally perceived usability, we surveyed age, sex, media usage, health literacy, and native language. Information about age and sex were part of the app-taken medical history. In addition to the SUS, we asked patients about which digital media tools were available to them in everyday life (possible answers: cell phone/smartphone, computer/laptop/notebook, tablet, television, none, and others; multiple answers were possible) and how many hours a day they used digital media (possible answers: 0-≤1, 1-≤2, 2-≤3, 3-≤4, or 4 or more hours). We asked three questions concerning health literacy as a proxy for education attainment, given that educational achievement is the central determinant of health literacy [28]. Questions covering the three aspects of finding/accessing, evaluating/appraising, and understanding health-related information and content were derived from the European health literacy survey [29,30] adapted for the German language (HLS-GER 2 [31]). The HLS-GER 2 uses a predefined 4-point Likert scale.

### Statistical Analysis

Data from the app were saved into a database and subsequently exported to a tab separated format for further analyses. Participants with two or more missing values of the SUS questionnaire were excluded from statistical analysis. In the case of one missing SUS response, we substituted the missing value with a neutral score of 2, as this method has been used with the SUS in previous research [32].

Sociodemographic data are presented as number and percentage of patients for each categorical data point. Mean and SD were utilized for interval or ratio-scaled data, which has become a common industrial goal. Sociodemographic data were compared between participants with SUS scores <80 and ≥80 using the Fisher exact test for 2×2 tables or the Fisher-Freeman-Halton test for categorical variables and the Wilcoxon rank-sum test for continuous variables. A multiple linear regression was conducted using sex, age, native German language, health literacy score, media usage duration per day, sickness level of the participants, and number of stated complaints in the app as independent variables and the SUS score as the dependent variable. Additionally, the individual SUS items were compared according to sex, age (<65 years vs ≥65 years), German native language, and tablet usage with the Wilcoxon rank-sum test. Data are visually presented as boxplots and radar charts. All analyses were carried out using R (4.1.3 under a GNU license) with the packages fmsb [33], psych [34], tidyr [35], dplyr [36], and ggplot2 [37].

## Results

### Patient Characteristics

**Figure 2:**
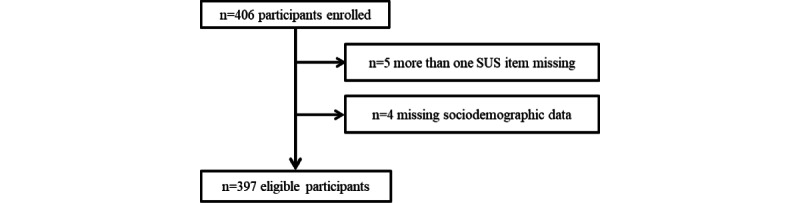
Flowchart of patient inclusion in the cross-sectional study capturing patients’ perceived usability of the app. SUS: System Usability Scale.

We aimed to include approximately 400 patients for this study. This target was set to be able to form subgroups and to ensure that all types of patient complaints were included in our sample, including those selected on a limited basis. In total, individual data from 397 participants were included, with 5 participants having one missing SUS item. Figure 2 shows the flowchart of included patients and Table 2 shows the patients’ characteristics. 

**Table 2:** Characteristics of the participants of the cross-sectional study capturing patients’ perceived usability of the app (N=397).

Characteristics			Value
**Sex, n (%)**			
	Female	225 (56.7)	
	Male	172 (43.3)	
Age (years), median (IQR)			35.0 (25.0)
**Age group (years), n (%)**			
	<30	152 (38.3)	
	30-65	223 (55.4)	
	65+	22 (6.3)	
Native language German, n (%)			328 (82.6)
**Devices used regularly^a^, n (%)**			
	Smartphone	389 (98.0)	
	Tablet	210 (52.9)	
	Computer/notebook	310 (78.1)	
	Television	296 (74.6)	
**Media usage duration per day (hours), n (%)**			
	<2	89 (22.4)	
	2-4	174 (43.8)	
	>4	134 (33.8)	
**Self-assessed health literacy, median (IQR)^b^**			
	Understanding doctor	2.0 (0.0)	
	Search and understand health information	2.0 (1.0)	
	Evaluate health information	1.0 (1.0)	
“**How sick do you feel?”^c^, n (%)**			
	I don’t feel sick	32 (8.1)	
	Just a little	70 (17.6)	
	Fairly	226 (56.9)	
	Very	61 (15.4)	
	Unbearably	6 (1.5)	

^a^Multiple selection possible.

^b^Measured on a 4-point (0-3) Likert-scale (higher scores indicate higher health literacy levels).

^c^Perceived severity of acute complaint.

### Usability for All Participants

**Figure 3:**
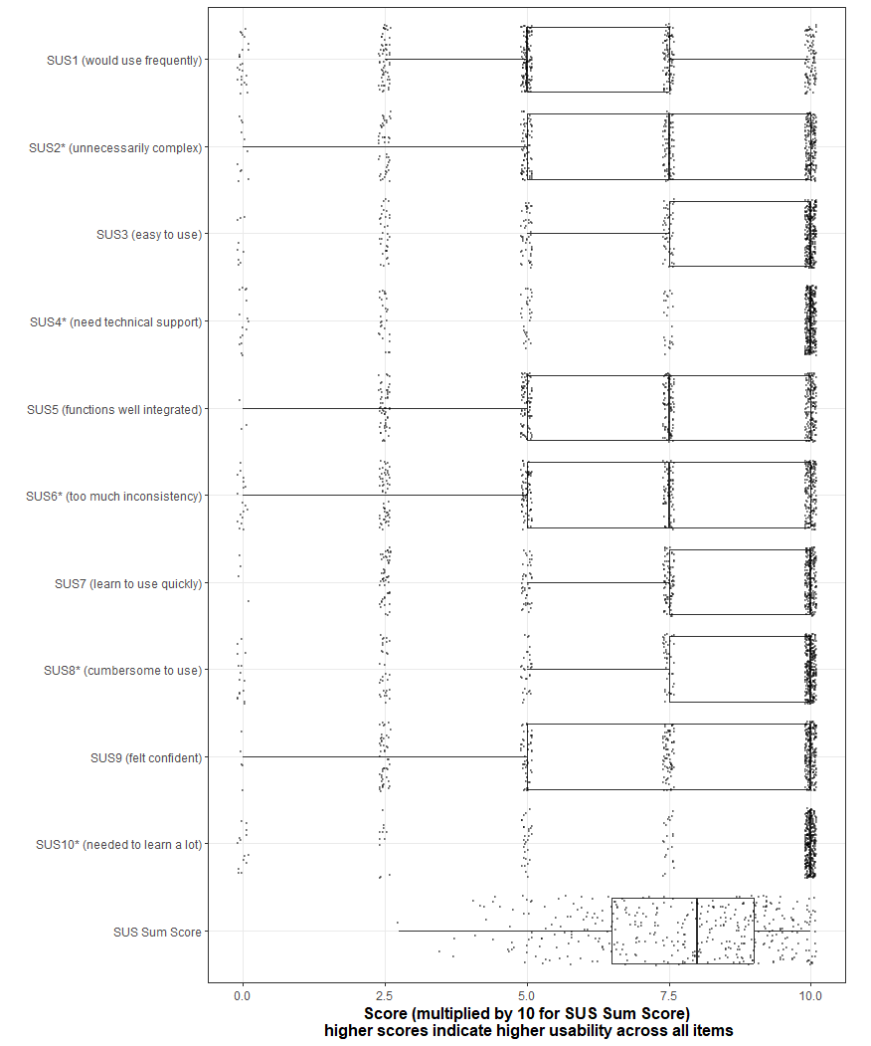
SUS items and sum scores for all study participants (N=397). SUS: System Usability Scale. *Scoring was inverted.

We found a mean total SUS score of 77.8 points, with 54.4% (216/397) of participants having SUS scores of 80 points or higher, indicating high usability of the app overall. Figure 3 shows boxplots of the individual items in which the scores were calculated for each statement. Irrespective of a positive or negative connotation, a higher score indicates a better result. The maximum score that can be achieved for each item is 10.

### Usability Stratified by Sociodemographic Factors

We divided the sample into two groups with the cutoff at a SUS score of 80. Participants with a SUS score of at least 80 were significantly younger, reported higher levels of technology device usage, and higher levels of self-assessed health literacy compared to participants with a SUS score below 80 (Table 3).

**Table 3:** Sociodemographic variables of study participants stratified by System Usability Scale (SUS) score.

Variable		SUS score<80 (n=181)	SUS score≥80 (n=216)	*P* value
**Sex, n (%)**				.09^a^
	Female	94 (51.9)	131 (60.6)	
	Male	87 (48.1)	85 (39.4)	
Age (years), median (IQR)		38.0 (26.0)	32.5 (22.0)	.002^b^
**Age group (years), n (%)**				.003^c^
	<30	55 (30.3)	97 (44.9)	
	30-65	111 (60.2)	112 (51.4)	
	65+	15 (9.4)	7 (3.7)	
Native language German, n (%)		142 (78.5)	186 (86.1)	.05^a^
**Devices used regularly^d^, n (%)**				
	Smartphone	174 (96.1)	215 (99.5)	.03^a^
	Tablet	84 (46.4)	126 (58.3)	.02^a^
	Computer/notebook	136 (75.1)	174 (80.6)	.22^a^
	Television	125 (69.1)	171 (79.2)	.03^a^
**Media usage duration per day (hours), n (%)**				.08^c^
	<2	48 (26.5)	41 (19.0)	
	2-4	69 (38.1)	105 (48.6)	
	>4	64 (35.4)	70 (32.4)	
**Self-assessed health literacy, median (IQR)**				
	Understanding doctor	2.0 (0.0)	2.0 (1.0)	.16^b^
	Search and understand health information	2.0 (0.0)	2.0 (1.0)	.01^b^
	Assess confidence of health information	1.0 (1.0)	1.0 (1.0)	.19^b^
“**How sick do you feel?”^e^, n (%)**				.35^c^
	I don’t feel sick	11 (6.1)	21 (9.7)	
	Just a little	30 (16.6)	40 (18.5)	
	Fairly	102 (56.4)	124 (57.4)	
	Very	34 (18.8)	27 (12.5)	
	Unbearably	3 (1.7)	3 (1.4)	

^a^Fisher exact test.

^b^Wilcoxon rank-sum test.

^c^Fisher-Freeman-Halton test.

^d^Multiple selection possible.

^e^Perceived severity of acute complaint.

A multiple linear regression predicting the SUS score was conducted, including sex, age, native German language, health literacy score, media usage duration per day, sickness level of the participants, and number of stated complaints in the app as independent variables (see Table 4). Age, sex, health literacy score, and German native language were significantly associated with SUS score. A higher age (t_385_=3.30, *P*=.001) and male sex (t_385_=1.98, *P*=.05) were negatively associated with SUS score, whereby a higher health literacy score (t_385_=2.83, *P*=.01) and German as a native language (t_385_=2.51, *P*=.01) were positively associated with SUS score. 

**Table 4:** Multiple linear regression predicting the System Usability Scale sum score.

Variable		β (95% CI)	*P* value
Male sex (reference=female)		–3.21 (–6.40 to –.02)	.05
age (per year)		–.17 (–.27 to –.07)	.001
German not native language (reference=yes)		–5.39 (–9.61 to –1.17)	.01
Does not use tablet (reference=yes)		–1.44 (–4.66 to 1.79)	.38
**Average daily media usage (reference=<2 h)**			
	2-4 h	3.21 (–.95 to 7.37)	.13
	>4 h	.01 (–4.46 to 4.48)	.99
Health literacy score (scale 0-9)^a^		1.48 (.45 to 2.51)	.01
How sick do you feel? (score 1-5)^b^		–1.05 (–2.92 to .82)	.27
Number of stated complaints (1-11)		–.98 (–2.11 to .15)	.09

^a^Higher scores indicate a higher level of health literacy.

^b^Perceived severity of acute complaint; higher values indicate a higher level of discomfort.

### Differences in Individual Items of the SUS

**Figure 4:**
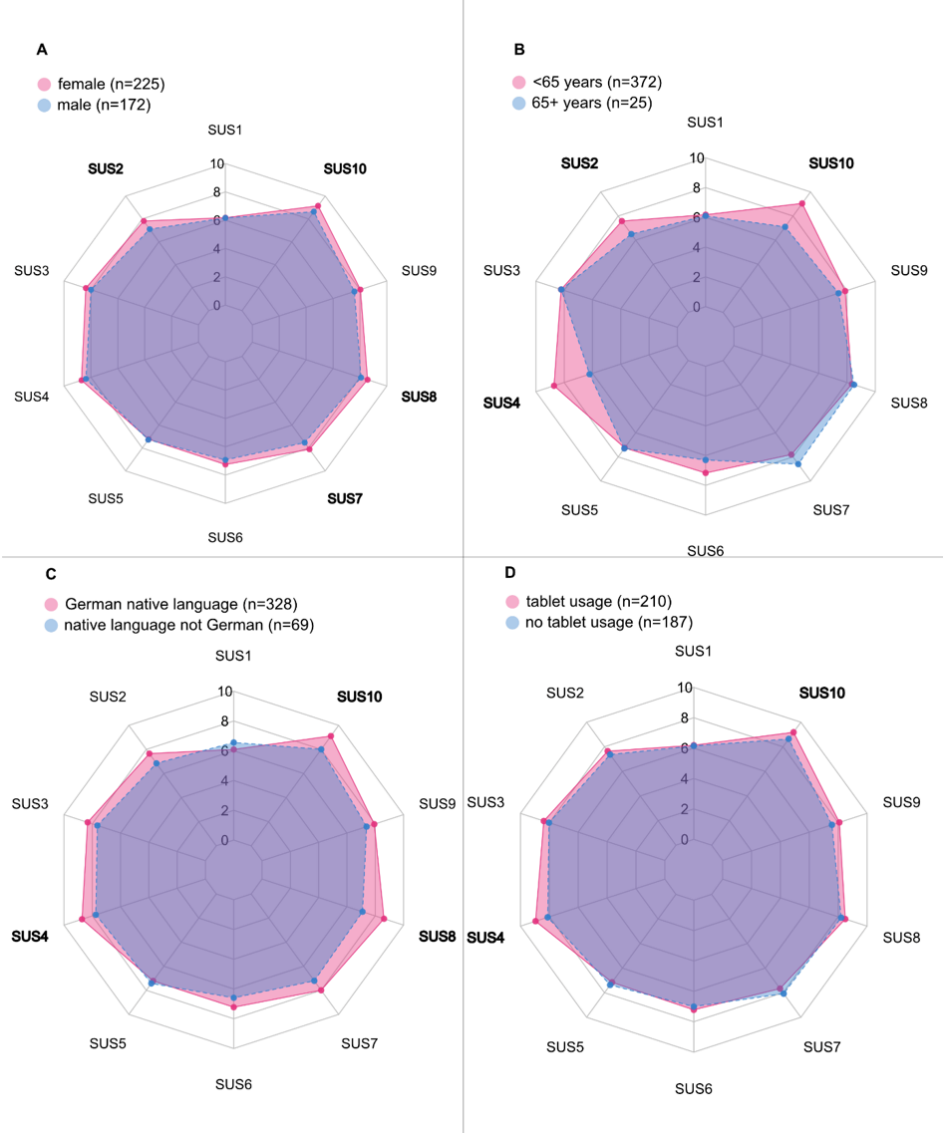
Mean System Usability Scale (SUS) score items (see Table 1) stratified according to sex (A), age (B), German as native language (C), and current tablet usage (D). bold: *P*<.05 (Wilcoxon rank-sum test).

Stratified according to sex, age, native language, and tablet usage (see Figure 4), significant differences were detected in SUS items 2 (“unnecessarily complex”), 4 (“need technical support”), 7 (“learn to use quickly”), 8 (“cumbersome to use”), and 10 (“needed to learn a lot”).

In comparing female and male respondents, all statements were rated more positively by female participants, except for items 1 (“would use frequently”) and 4 (“need technical support”). Female participants also scored significantly higher than male participants on items 2 (“unnecessarily complex”) (mean 7.82 vs 7.11; *P*=.02), 7 (“learn to use quickly”) (mean 8.11 vs 7.53; *P*=.05), 8 (“cumbersome to use”) (mean 8.57 vs 8.08; *P*=.05), and 10 (“needed to learn a lot”) (mean 9.14 vs 8.63; *P*=.03).

Respondents aged 65 years and older scored significantly higher on items 2 (“unnecessarily complex”) (mean 7.58 vs 6.50; *P*=.04), 4 (“need technical support”) (mean 8.72 vs 6.20; *P*<.001), and 10 (“needed to learn a lot”) (mean 9.05 vs 7.; *P*<.001) compared to their counterparts.

German native language speakers scored significantly higher on items 4 (“need technical support”) (mean 8.73 vs 7.75; *P*=.001), 8 (“cumbersome to use”) (mean 8.62 vs 7.10; *P*<.001), and 10 (“needed to learn a lot”) (mean 9.11 vs 8.01; *P*<.001) relative to nonnative speakers.

Lastly, patients who regularly use a tablet had significantly higher SUS scores on items 4 (“need technical support”) (mean 8.95 vs 8.11; *P*<.001) and 10 (“needed to learn a lot”) (mean 9.18 vs 8.64; *P*=.02) in comparison to those of participants who reported reduced levels of tablet use.

## Discussion

### Principal Findings

In this study, we evaluated the usability of an app in taking medical histories in general practice directly from patients using the SUS [19].

The app achieved a mean SUS score of 77.9, which corresponds to a B+ grade on the curved grading scale [27] and represents a “better” product that does not necessarily need improvement [25]. Other medical devices, even those widely used at home, have lower SUS scores. Kortum and Peres [38] assessed the usability of home health care devices among students, thus representing relatively young, healthy, and well-educated participants. SUS scores for these devices ranged from 65 for an epinephrine injector to 67 for a pregnancy test kit and 81 for a thermometer, even with previous experience using these devices.

To ensure patients can be active participants in the digital medical history–taking process, the app must be easy and intuitive to use without technical introduction or support. This importance is reflected in items 3 (“easy to use”), 4 (“need technical support“), 7 (“learn to use quickly”), and 10 (“needed to learn a lot”). Mean values between 7.8 and 8.9 for these items indicate that intuitive use has been successfully addressed in the development of our app. Item 1, assessing the frequency of app usage, scored the lowest (6.2), which can be explained by the app’s implementation solely in a medical setting and not utilized regularly in leisure time. As such, this finding is the least meaningful for our purpose.

In a pilot study by Melms et al [39], a self-completed tablet-based digital questionnaire designed for collecting medical histories in an emergency department was found to score high with respect to perceived usability. The design and content were similar to those of our app; however, their questions were only based on the SUS, which does not allow direct comparison. Other comparable instruments, although also for emergency departments, have been tested for usability in pilot studies using self-developed satisfaction surveys [40,41], a single question, and researcher or staff documentation of a patient’s need for assistance [42]. In these studies, patients were mostly satisfied with the self-administered medical history–taking tools and reported good ease of use. Taken together, these results give hope that it is possible to design a medical history app that is perceived as user-friendly.

Nonetheless, obstacles to implementing a digital tool in general practice settings can be multifaceted. Surprisingly, we found that sex was significantly associated with usability; female participants had significantly higher SUS scores than male participants. The fact that men scored higher than women for item 4 (“need technical support”) suggests that men felt more confident than women with using the app. Previous studies demonstrated that men tend to report overconfidence in their abilities, especially in fields with a male connotation [43], which computer science certainly represents [44]. Therefore, it is unclear whether men really would have needed less help or whether they overestimated themselves in their technical skills.

Our study suggests that older people are more likely to have difficulties with the handling of such an app. This aligns with a study showing that from the retirement age of 65 years, digital media use among the German population begins to decline dramatically [45] and a positive attitude toward digitalization decreases with increasing age [46]. Older age has a negative impact on the broad usability score given to a user interface [25]. To that end, this study cannot definitively conclude if the older participants of this study actually perceived the app to be of relatively low usability or if their more negative attitude toward the benefit of new technologies prompted them to give lower scores. Due to the small sample size of participants aged 65 years and older, it is not possible to assert how older people in general would cope with the handling of the app. Since GPs are consulted predominantly by older people [47], further research should focus on app testing with older patients to obtain specific feedback, including suggestions for improvement.

Having learned German as a native language was positively associated with a higher SUS score, although only patients with sufficient German language proficiency were included in the study [17]. This could be due to two different reasons: despite the app’s plain language, it is possible that some of the medical history questions or SUS items were not understood properly.

Daily media use was not associated with the SUS score, which suggests that the app is designed to also ensure that people with limited digital experience do not feel overstrained with its operation.

### Limitations

Despite our efforts, this study comes with several limitations. The number of older participants (ie, aged 65 years and above) was relatively low in comparison to their constituents in GP practice settings [47]. One potential reason could be a more pronounced skepticism toward digital tools in older generations, leading to an increase in refusal for participation in the study among older patients. However, as no screening lists were maintained, this is mere speculation. A screening list should be obtained in future studies to be able to characterize individuals who declined participation. Another consideration is that people with lower levels of digital media literacy use may not have agreed to participate in the study.

Data collection was performed during the SARS-CoV-2 pandemic, which may have disproportionately impacted study participants as certain patient groups may have avoided seeing a doctor or were more likely to refuse to participate in the study to avoid unnecessary contact. This could have included especially vulnerable groups such as older people or those with multimorbid conditions.

The Likert scale of the SUS questions shown with clickable singular dots was replaced by a slider on December 8, 2021. In the dot-based representation, it was compulsory to make an entry before continuing, whereas the slider was automatically set to the neutral center and could be shifted. This may have led to incorrectly rated items. For example, this may have occurred in instances of the internet faltering or the patient having double-clicked without noticing. Since there were repeated questions about the word “Inkonsistenzen” (SUS item 2), we replaced it by the more common synonym “Unstimmigkeiten” (English translation: discrepancies). Furthermore, hardware as well as the operating system may have influenced the evaluation of the personally perceived usability of a system [48]. For this study, iPad Minis with the iOS operating system were exclusively used. Therefore, possible differences in assessment related to the operating system and hardware are not part of this study.

The SUS is able to classify the usability of a system but is unable to identify specific usability issues nor capture the usability of the system in its entirety. For a more in-depth usability evaluation, different methods could be used (eg, interviews and observations). During data collection, staff were able to observe usability problems. In their observations, multiple-choice, single-choice, and hybrid questions as well as the slider did not appear to cause any difficulties. In contrast, problems concerning the handling of the app arose when participants were required to input free-text entries (eg, age, height, weight). Further, some study participants were unclear on how to open and close the on-screen keyboard. Some participants also did not understand that the figure on which a pain or an injury could be assigned to a body region (see Figure 1E) could be rotated by clicking on an icon at the bottom left of the screen. This means that, for example, back pain may have been falsely reported as abdominal pain. Lastly, an unstable internet connection arose during data collection, which caused the app to be unresponsive intermittently. These factors may have influenced the SUS score.

### Conclusion

The app examined in this study for medical history taking passes the usability test based on the SUS and appears to function on par with other digital tools that have become well-integrated in our everyday lives. However, not all people adapted equally well to the app. For successful implementation, all end users, regardless of age, technical affinity, health literacy, or preferred language, must be able to use such a tool. Only if that is attained, providing practical digital solutions can contribute to the efficient and effective delivery of health care services. Therefore, further research should focus on the identification of causes for difficulties of using the app as well as finding appropriate solutions.

## Appendix

Multimedia Appendix 1 Original and German versions of the System Usability Scale (SUS) and the items customized for this study.

## Abbreviations

DASI: digitally assisted information acquisition before medical consultation
GP: general practitioner
HLS-GER 2: European health literacy survey adapted for the German language
SUS: System Usability Scale

## Data Availability

The data sets used and analyzed during the study are not publicly available due to the decision of the research ethics board but can be obtained from the authors upon reasonable request within a data sharing agreement.
