# Supplementary material for: Usability of an App for Medical History Taking in General Practice From the Patients’ Perspective: Cross-Sectional Study
Source: JMIR Hum Factors. 2024 Jan 5;11:e47755. doi: 10.2196/47755 (PMC10799287; doi:10.2196/47755)
Supplement: Multimedia Appendix 1 [file humanfactors_v11i1e47755_app1.docx]

**Multimedia Appendix 1.** Original and German versions of the System Usability Scale (SUS) and the items customized for this study.

| **SUS English [19]** | **SUS German [26]** | **Modified items German** |
| --- | --- | --- |
| I think that I would like to use this system frequently. | Ich denke, dass ich dieses Produkt häufig verwenden möchte. | Ich denke, dass ich die App regelmäßig nutzen werde. |
| I found the system unnecessarily complex. | Ich fand das Produkt unnötig komplex. | Ich empfinde die App als unnötig komplex. |
| I thought the system was easy to use. | Ich dachte, das Produkt war einfach zu bedienen. | Ich empfinde die App einfach zu nutzen. |
| I think that I would need the support of a technical person to be able to use this system. | Ich denke, dass ich die Unterstützung einer technischen Person brauche, um dieses Produkt nutzen zu können. | Ich denke, dass ich technischen Support brauchen werde, um die App zu nutzen. |
| I found the various functions in this system were well integrated. | Ich fand, die verschiedenen Funktionen in dem Produkt waren gut integriert. | Ich finde, dass die verschiedenen Funktionen in der App gut integriert sind. |
| I thought that there was too much inconsistency in this system. | Ich dachte, dass dieses Produkt nicht konsistent genug war. | Ich finde, dass es in der App zu viele Inkonsistenzen/Unstimmigkeiten gibt.^*^ |
| I would imagine that most people would learn to use this system very quickly. | Ich würde mir vorstellen, dass die meisten Leute sehr schnell lernen würden, dieses Produkt zu benutzen. | Ich kann mir vorstellen, dass die meisten Menschen die App schnell zu beherrschen lernen. |
| I found the system very cumbersome to use. | Ich fand dieses Produkt sehr umständlich zu benutzen. | Ich empfand die Bedienbarkeit als sehr umständlich. |
| I felt very confident using the system. | Ich habe mich sehr selbstsicher gefühlt, dieses Produkt zu verwenden. | Ich habe mich beim Einsatz von der App sehr sicher gefühlt. |
| I needed to learn a lot of things before I could get going with this system. | Ich musste eine Menge Dinge lernen, bevor ich mit diesem Produkt loslegen konnte. | Ich musste eine Menge Dinge lernen, bevor ich mit der App arbeiten konnte. |

* During the data collection, it was noted that some participants had problems understanding the word “Inkonsistenzen” (English: inconsistency) in the sixth item of the SUS. Therefore, we decided to replace the word by the simpler synonym “Unstimmigkeiten” (English: discrepancies).
